# Influence of the Temperature-Dependent Characteristics of CFRP Mechanical Properties on the Critical Axial Force of Drilling Delamination

**DOI:** 10.3390/polym15030680

**Published:** 2023-01-29

**Authors:** Hongxiao Wang, Yahang Wu, Yi Zhang, Xiaohui Zhang

**Affiliations:** 1 School of Mechanical and Electrical Engineering, Henan University of Technology, Zhengzhou 450001, China; 2 School of Mechanical Engineering, Xi’an Jiaotong University, Xi’an 710049, China

**Keywords:** CFRP drilling, drilling delamination damage, critical force, temperature-dependent characteristics of CFRP mechanical properties

## Abstract

Previous studies have often assumed that the mechanical properties of Carbon Fibre Reinforced Plastics (CFRP) remain unchanged during drilling. In fact, due to the increase in drilling temperature, the mechanical properties of the composites change greatly, and this then affects the critical force. In addition, previous studies have often assumed that the failure mode of CFRP drilling was a type I crack failure. In fact, due to the complexity of the CFRP drilling process, the failure modes are often coupled with different failure modes, so type I cracks alone cannot reflect the actual cracking situation. Therefore, a three-dimensional drilling Finite Element Modeling (FEM) was established to analyze the failure modes of CFRP drilling delamination, and the I/III mode was determined; then, a new drilling critical force mechanics model, which considers the temperature dependence of CFRP mechanical properties and the failure modes of CFRP drilling delamination, was established based on the classical drilling critical force mechanics model; the results of the mechanics model were validated by drilling critical force experiments under different temperatures. The effects of the temperature dependence of CFRP mechanical properties on the drilling critical force were investigated and analyzed.

## 1. Introduction

In recent years, carbon fiber-reinforced polymer composites (CFRP) have been widely used in aviation, aerospace, subway tracks, and other fields. However, due to their anisotropy, multiphase, and heterogeneous nature, they are prone to drilling delamination damage in the drilling process, which seriously affects the reliability of CFRP parts [1]. To reduce the delamination damage in CFRP drilling, scholars have conducted a lot of research on the delamination damage in the drilling process around the drilling force. It is generally believed that there is a critical axial force (*P_C_*) [2]. When the drilling axial force is lower than *P_C_*, delamination damage will not occur during drilling; on the contrary, delamination damage will occur when the force is higher than *Pc* [3]. Based on these studies, a series of analytical models for predicting the *P_C_* has been established [4]. For example, Hocheng et al. [5] proposed the concept of *P_C_* in 1990 and established the mechanical analytical model of *P_C_* based on linear elastic fracture mechanics and classical beam–plate bending theory. They believe that delamination defects do not occur when the axial force is less than a certain critical value in the process of composite drilling. However, their model assumes that the composite material is isotropic, which is inconsistent with the actual situation of the material, resulting in large errors when predicting the *P_C_*.

Since then, many scholars have improved the model of *P_C_* based on the Hocheng model. For example, Lachuad et al. [6] established an analytical model of *P_C_* with anisotropic mechanical properties of composites based on the classical laminated plate theory and Hocheng model, and they proved that the analytical model established by distributed load has higher accuracy than the concentrated load model for the first time. Jain et al. [7] first introduced the idea that the shape of the drilling delamination area is elliptic and characterized the delamination area by a coefficient, which improved the prediction accuracy of the *P_C_* analytical model. However, neither the model of Lachuad [6] nor Jain [7] considers the bending torsion coupling effect of laminates. Therefore, their models can only predict the *P_C_* of unidirectional laminated plates. Zhang et al. [8] considered the bending torsion coupling effect of laminates based on Jain [7] and established an analytical model of *P_C_* that can analyze any laminate stack sequence. To further improve the accuracy of *P_C_*, Ojo et al. [4] subdivided the chisel edge and the main cutting edge of a drill. They assume that the force caused by the chisel edge is a concentrated force and that the force caused by the main cutting edge is a uniformly distributed force. In their model, they put forward the hypothesis that the concentrated force and the uniformly distributed force both exist, and they confirmed that the ratio of concentrated force and distributed force has an impact on the *P_C_*. In addition, the model also puts forward the assumption that the drilling delamination crack propagation form is I/II mixed type and analyzes it. Saoudi et al. [9] first studied the analytical model of the *P_C_* under mechanical–thermal coupling. The results showed that the drilling *P_C_* would change under the influence of drilling temperature, but the mechanical properties of the CFRP materials were not considered in the model. Table 1 is a summary of the *P_C_* model in the above references, where *P_C_* is the critical axial force, *G_IC_* is a mode I crack, and *C*_3,_ *K*, *D*_11_, *D*_22_, and *D* are the stiffness coefficients of undrilled materials.

Through the above analysis of the *P_C_* model, it can be found that the “fracture toughness” is an important factor that affects the *P_C_*. However, due to the complexity of the CFRP drilling process, it is difficult to directly observe the failure mode of drilling delamination from the experimental results. Most of the above models assume that the failure mode of drilling delamination crack is the mode I crack failure form. In fact, due to the complexity of force in the drilling process of composite materials, the failure mode is often a combination of different failure modes [1], and thus, using the mode I crack alone cannot reflect the actual cracking situation. In addition, for CFRP, because the temperature has a great impact on the mechanical properties of the resin matrix, some mechanical properties related to the resin, especially the fracture toughness *G_IC_* or *G_I__I__C_*, will change greatly with an increase in temperature, but the above models do not consider the influence of the temperature-dependent characteristics of CFRP mechanical properties on *P_C_*. This research found that *G_IC_*, *C*_3_, *K*, *D*_11_, *D*_22_, and *D* in the classical calculation equation of *P_C_* are affected by temperature changes and that the influence trends are different. The drilling process of composite materials produces a lot of heat, which increases the drilling temperature. Therefore, the influence of drilling temperature on the *P_C_* should also be considered when analyzing the *P_C_* of CFRP drilling delamination.

Therefore, we established a three-dimensional finite element drilling model of CFRP, including an interface phase, to study the interlaminate damage mechanism of CFRP drilling and obtain the interlaminate damage mode of CFRP caused by drilling; then, based on the *P_C_* model of Zhang [8], we deduced a new *P_C_* model that considers the temperature-dependent characteristics of CFRP mechanical properties and the damage mode of CFRP interlaminate; finally, the *P_C_* model was verified by static compression experiments at different temperatures.

## 2. Finite Element Simulation Analysis of CFRP Three-Dimensional Drilling

### 2.1. Material Constitutive Definition

CFRPs are defined as anisotropic linear elastic constitutive materials, and the failure criterion adopts the three-dimensional Hashin failure criterion [10,11]. As the damage criterion that comes with Abaqus does not have a three-dimensional Hashin failure criterion, the FORTRAN language must be used to write the three-dimensional Hashin failure criterion and damage evolution into the user material subprogram VUMAT for analysis and calculation. The interlamination interface is established by cohesive elements. Because the interlamination thickness of the composite material is very thin, the zero-thickness cohesive element is used to model it in the simulation analysis. The damage criterion adopts the traction–separation law with the I/II/III mixed modes [12,13,14]. The initial failure mode adopts the secondary stress failure mode, and the damage evolution process adopts the energy-based Benzeggagh–Kenane (BK) damage evolution criterion [15]. The damage evolution process after cracking adopts the energy-based BK damage evolution criterion:

(1) $$G_{IC}+\left(G_{IIC}-G_{IC}\right){\left\{\frac{G_{II}+G_{III}}{G_{I}+G_{II}+G_{III}}\right\}}^{\eta }=G_{c}$$

where η is the energy index; *G_IC_*, *G_IIC_*, and *G_IIIC_* are the fracture toughness (N/mm) of the three cracking modes, respectively, and *G_I_*, *G_II_*, and *G_III_* are the energy release rates during calculation (N/mm).

### 2.2. Finite Element Modeling and Parameter Setting

A carbide twist drill with a diameter of 8 mm is introduced into Abaqus, and the elastic modulus of the drill material is set to 640,000 Mpa and the Poisson’s ratio to 0.22. As the elastic modulus of the tool material is much larger than that of CFRP, to save calculations during the analysis, we set the drill as a rigid body. To define the material properties and directional properties of the composite material, CFRP adopts T300/epoxy resin, the fiber volume fraction is about 62% according to Hengshen Co. Ltd., Zhenjiang, China, the layering method is [0/45/90/−45]s, and the thickness of a single layer is 0.125 mm, totaling 1 mm. The element type is 3D stress. The material properties are shown in Table 2, which were provided by Hengshen Co. Ltd., China. The interlamination interface adopts a zero-thickness cohesive element, and the material properties are shown in Table 3.

**Figure 1:**
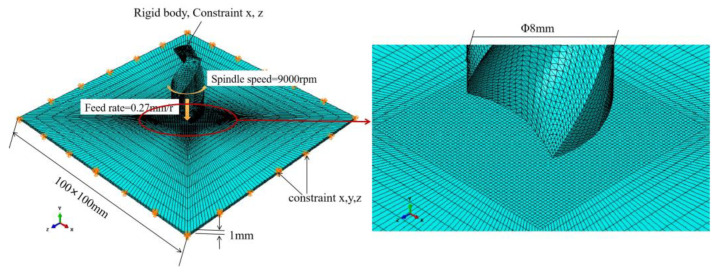
CFRP three-dimensional drilling finite element model.

During the simulation calculation, the X-axis, Y-axis, and Z-axis movement freedom around the CFRP plate is constrained, and the downward feed of the drill bit and the rotational speed of the spindle rotating around the drill bit are defined. To reduce the calculation time and increase the degree of delamination damage, a larger feed is selected in the simulation. The feed rate and spindle speed are 0.27 mm/r and 9000 rpm, respectively. The downward velocity along the Y-axis is set to 40.5 mm/s. The rotation speed around the Y-axis is set to 942 rad/s. At the same time, the movement freedom of the rigid body reference point of the drill’s X-axis and Z-axis is constrained. The cutting edge of the drill is in contact with the composite material. Due to the anisotropy of CFRP, its friction coefficient will vary with the cutting angle, ranging from 0.3 to 0.8 [16]. As the purpose of finite element analysis in this study is to observe the three-dimensional drilling process, the intermediate value of 0.5 is selected as the friction coefficient between the drill bit and the composite material. The established CFRP three-dimensional drilling finite element model is shown in Figure 1.

## 3. Analysis of Simulation Results

**Figure 2:**
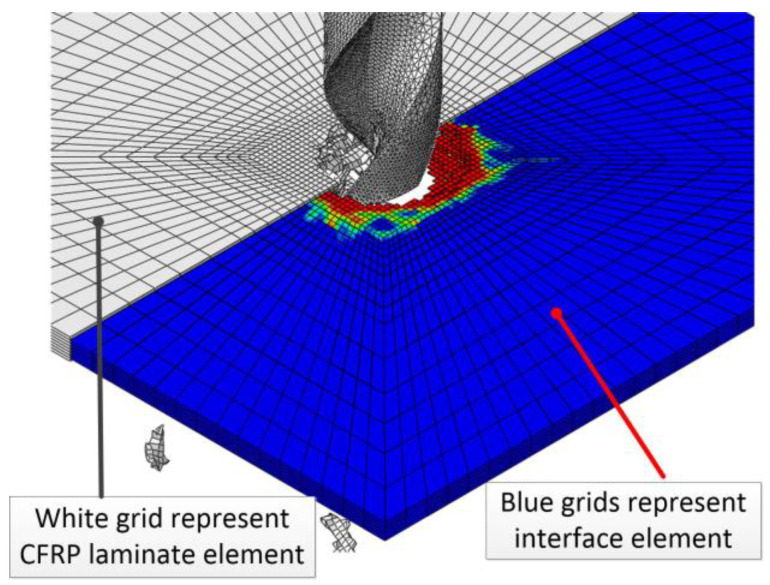
Finite element simulation results of CFRP three-dimensional drilling.

Figure 2 shows the simulation results of CFRP drilling. The white part is the CFRP element, the blue part is the interlamination interface element, and the red part represents the damage degree of the interface layer. To more clearly show the position and failure mode of the interlamination element in the laminated plate during the drilling process, some composite elements are hidden in Figure 2.

**Figure 3:**
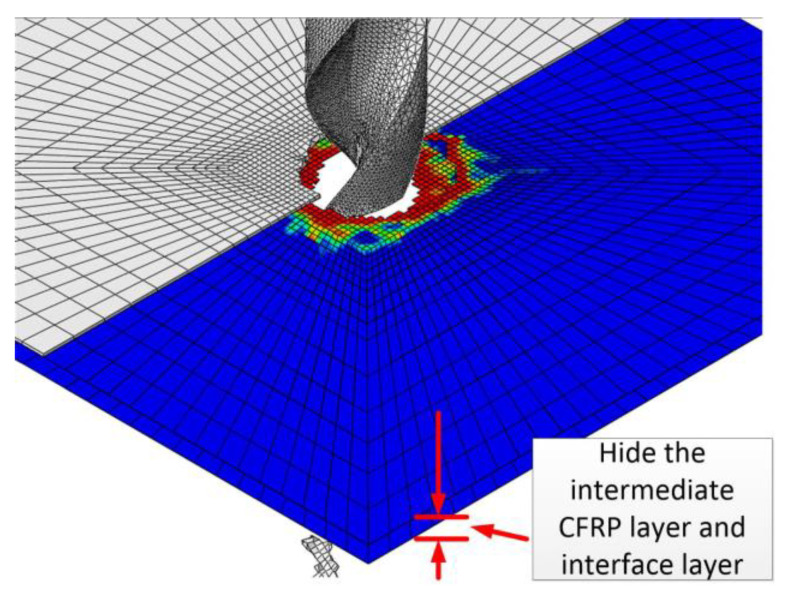
Comprehensive diagram of Figure 4.

**Figure 4:**
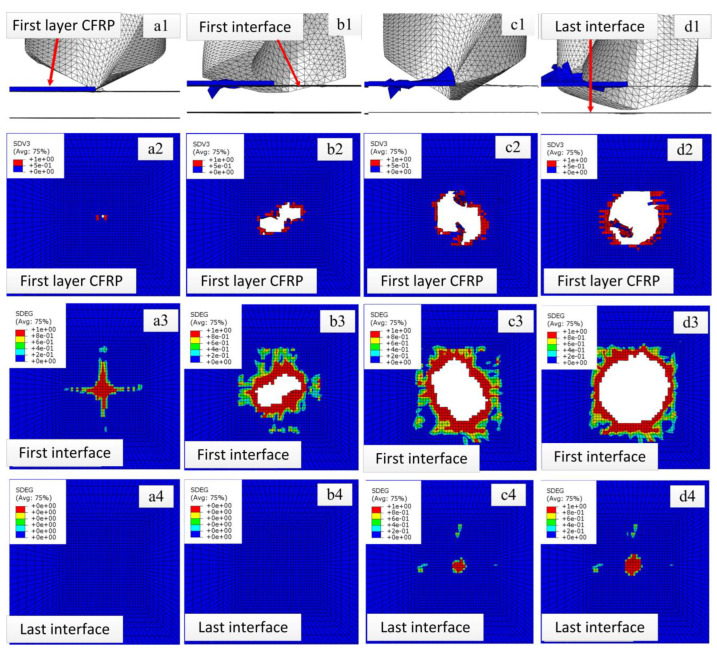
Representative figures of drill bit passing through every layer of CFRP and every layer interface of drilling simulation results at different stages. (**a1**–**a4**) Drilling stage I. (**b1**–**b4**) Drilling stage II. (**c1**–**c4**) Drilling stage III. (**d1**–**d4**) Drilling stage IV.

In order to more clearly show the damage and failure process of interlaminate interface elements during the drilling process, part of the CFRP element layer and the interlaminate element layer of the middle part are hidden in Figure 3 and Figure 4, and only the first layer of CFRP, the first layer of interlaminate interface elements (short for first interface), and the last layer of interlaminate interface elements (short for last interface) are reserved for location reference. Figure 3 is a comprehensive diagram of Figure 4. Figure 4 is a representative figure of the drill bit passing through every layer of CFRP and every layer of the interface at different stages. Figure 4(a1–d1) is the front view of Figure 4 (a2–a4), (b2–b4), (c2–c4), and (d3,d4), respectively.

It can be seen from Figure 4(a1–a4) that, when the drill bit is drilled to stage I, the chisel edge makes contact with the first layer of the CFRP. Although the removal amount of CFRP material in the first layer is very small Figure 4(a2), the interlaminar element in the first layer also begins to be damaged due to the downward bending load. When the drill bit is drilled to stage II Figure 4(b1–b4), the main cutting edge starts to work, the contact delamination area between the first layer of the CFRP and the drill increases Figure 4(b2), and the damage delamination area of the first interface increases Figure 4(b3). At this time, the last interface is still not affected. When the drill bit is drilled to stage III Figure 4(c1–c4), the main cutting edge starts working, the drilling axial force increases, the contact delamination area between the first layer of the CFRP and the drill increases Figure 4(c2), and the damage delamination area of the first interface increases Figure 4(c3). At this time, the last interface begins to be damaged Figure 4(c4). When the drill bit is drilled to stage IV Figure 4(d1–d4), the main cutting edge is fully working, and the drilling axial force increases to its maximum. Accordingly, the damage delamination area of both the first layer of the CFRP and the first interface increase to their maxima Figure 4(d2,d3). As the drill bit is not in contact with the last interface, the damage evolution area of the last interface at this stage does not expand significantly. The above results show that the [0/45/90/−45]s layers can cover all the drilling processes of this bit, and all CFRP layers and interface layers need to go through stages I to IV.

**Figure 5:**
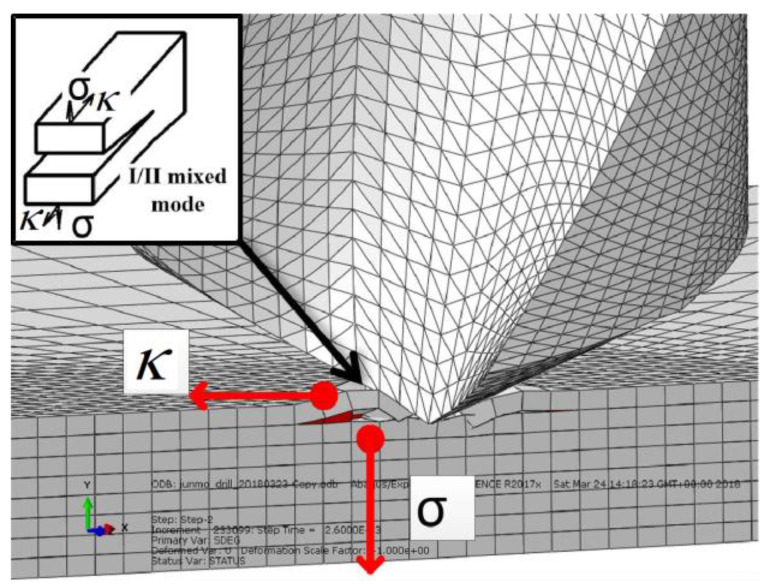
Crack form of drill entry chisel edge.

Due to the laminated structure characteristics of CFRP, the crack form often occurs between layers during drilling, accompanied by a coupling effect. To judge the cracking form of the laminated plate in the drilling process, it is necessary to analyze the interlamination failure form in FEM results combined with different drilling stages. Figure 5 shows the section view of the finite element simulation results when the chisel edge just contacts the material (drilling stage I). It can be seen from Figure 5 that due to the width of the chisel edge, it will exert a pressing force on the vertical feeding direction of the first layer of the CFRP material of the laminate, which will cause the material of this layer to produce a shear force (κ) in the vertical extrusion direction and then cause a type II crack. At the same time, the downward feeding movement will produce a downward extrusion force (*σ*) on the uncut layer and then cause a type I crack. As the shear force and extrusion force exist simultaneously, the CFRP at the entrance is affected by the chisel edge, resulting in the coupling of I/II cracks (Figure 5).

**Figure 6:**
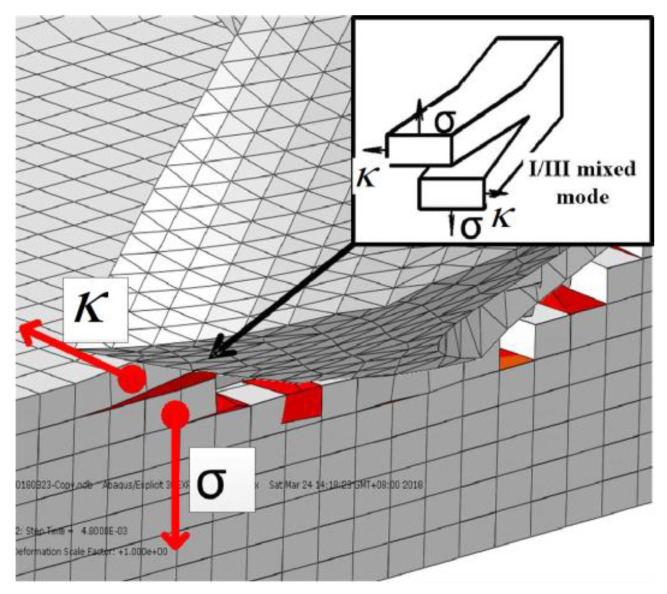
Crack form of main cutting edge at drilling inlet.

When the drill bit continues to feed along the axial direction, the main cutting edge begins to work (drilling stage II). Any point on the main cutting edge will generate a shear force (κ) in the direction of the cutting speed, which will form a type III crack. At the same time, the chisel edge feeds downwards, and the downward feeding movement will produce a downward extrusion force (*σ*) on the uncut layer, causing a type I crack. Because the shear force in the direction of the cutting speed and the downward extrusion force exist simultaneously, this results in the coupling of I/III cracks (Figure 6).

**Figure 7:**
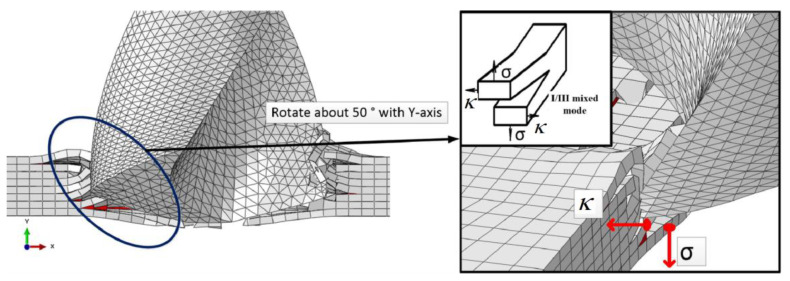
Crack form of main cutting edge at drilling exit.

When the main cutting edge is fully working (drilling stage IV), the material removal form and the delamination form caused by the chisel edge and the main cutting edge are the same, type I/II cracks are caused by the chisel edge, and type I/III cracks are caused by the main cutting edge. However, when drilling to the exit, as the number of uncut layers decreases, the residual stiffness also decreases, and the I/II cracks caused by the chisel edge are removed by the movement of the main cutting edge. Because the shear force (κ) in the direction of the cutting speed and the downward extrusion force (*σ*) exist simultaneously, I/III cracks couple at the drilling exit. Therefore, the main delamination form of the drilling exit is the I/III mixed crack (Figure 7).

## 4. Modeling of CFRP Drilling Delamination *P_C_*

**Figure 8:**
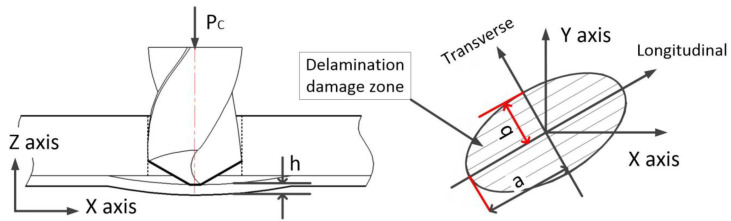
Force analysis of delamination damage at the exit of CFRP drilling.

In this section, based on the drilling axial force model of Zhang [8], the temperature-dependent characteristics of CFRP mechanical properties and the mixed I/III delamination failure mode at the drilling exit, a prediction model of the drilling *P_C_* is established. The model derivation process is as follows:

First, assuming that the shape of the CFRP drilling exit layer is elliptical [5], the force state of the delamination damage at the drilling exit is shown in Figure 8. In Figure 8a, *h* is the thickness of the undrilled layers, *a* is the longitudinal radius of the ideal damaged ellipse, and b is the transverse radius of the ideal damaged ellipse (Figure 8b).

Assuming that all the work accomplished via the drilling axial force at the drilling exit (Figure 8) is converted into energy released by delamination and energy required by material strain, then, based on the linear elastic fracture mechanics and the law of energy conservation, the balance relation equation of energy required for delamination can be established:

(2) $$P_{C}d\omega_{0}=G_{C}dA+dU$$

where *Pc* is the critical axial force (N); *dω*_0_ is the differential cross-section deflection of uncut laminates; *G_C_* is the critical energy release rate (N/mm); *dA* is the differential layered area (mm); and *dU* is the differential of the strain energy required for the elastic strain of the material (N).

The process of solving the *Pc* is also the process of solving *dω*_0_, *G_C_*, *dA*, and *dU* in the equation. In previous studies, the influence of cutting temperature has not been considered when solving the above parameters. This study analyzes the equation based on the temperature-dependent characteristics of CFRPs’ mechanical properties. The detailed process is as follows.

### 4.1. The Solution of Section Deflection dw_0_ of Undrilled Layer Material

According to the classical theory of laminated plates, the constitutive relationship of CFRPs considering the mechanical and thermal coupling properties is shown in Equation (3).

(3) $$\left[\begin{array}{l} N_{x}\\ N_{y}\\ N_{xy}\\ M_{x}\\ M_{y}\\ M_{xy} \end{array}\right]=\left[\begin{array}{cccccc} A_{11} & A_{12} & A_{16} & B_{11} & B_{12} & B_{16}\\ A_{12} & A_{22} & A_{26} & B_{12} & B_{22} & B_{26}\\ A_{16} & A_{26} & A_{66} & B_{16} & B_{26} & B_{66}\\ B_{11} & B_{12} & B_{16} & D_{12} & D_{12} & D_{16}\\ B_{12} & B_{22} & B_{26} & D_{12} & D_{22} & D_{26}\\ B_{16} & B_{26} & B_{66} & D_{16} & D_{26} & D_{66} \end{array}\right]\left[\begin{array}{l} \varepsilon_{x}\\ \varepsilon_{y}\\ \varepsilon_{xy}\\ \tau_{x}\\ \tau_{y}\\ \tau_{xy} \end{array}\right]=\left[\begin{array}{l} N_{x}^{m}\\ N_{y}^{m}\\ N_{xy}^{m}\\ M_{x}^{m}\\ M_{y}^{m}\\ M_{xy}^{m} \end{array}\right]+\left[\begin{array}{l} N_{x}^{T}\\ N_{y}^{T}\\ N_{xy}^{T}\\ M_{x}^{T}\\ M_{y}^{T}\\ M_{xy}^{T} \end{array}\right]$$

where [*N_i_*] is the total internal force under mechanical–thermal coupling; [*M_i_*] is the total bending moment under mechanical–thermal coupling; [*N_i_^m^*] is the internal forces caused by the mechanical load; [*M_i_^m^*] is the bending moments caused by mechanical forces; [*N_i_^T^*] is the internal forces caused by thermal loads; [*M_i_^T^*] is the bending moment induced by the thermal load; [*A_i_*] is the components of the extensional stiffness matrix; [*B_i_*] is the components of the extension–bending coupling matrix; and [*D_i_*] is the components of the bending coupling matrix. The calculation methods of [*A_i_*], [*B_i_*], and [*D_i_*] are from [8].

Assuming that the displacement is very small during drilling, the relationship between strain, curvature, and displacement is:

(4) $$\varepsilon_{x}=\frac{\partial u}{\partial x}$$

(5) $$\varepsilon_{y}=\frac{\partial v}{\partial y}$$

(6) $$\varepsilon_{xy}=\frac{\partial u}{\partial x}+\frac{\partial v}{\partial y}$$

(7) $$\tau_{x}=-\frac{\partial^{2}\omega }{\partial x}$$

(8) $$\tau_{y}=-\frac{\partial^{2}\omega }{\partial y}$$

(9) $$\tau_{xy}=-2\frac{\partial^{2}\omega }{\partial x\partial y}$$

where *ε* is the in-plane strain; u and v are the displacements in the x and y directions, respectively; *τ* is the curvature of the laminate midplane; and *ω* is the displacement perpendicular to the laminate.

The drilling force model of undrilled CFRP laminate can be simplified as a plate model with a concentrated force in the middle. According to the basic bending equilibrium differential equation of plate shell theory:

(10) $$\frac{\partial N_{x}}{\partial x}+\frac{\partial N_{xy}}{\partial y}=0$$

(11) $$\frac{\partial N_{y}}{\partial y}+\frac{\partial N_{xy}}{\partial x}=0$$

(12) $$\frac{\partial^{2}M_{xx}}{\partial x^{2}}+2\frac{\partial^{2}M_{xy}}{\partial x\partial y}+\frac{\partial^{2}M_{yy}}{\partial y^{2}}+q=0$$

Assuming that the load is uniformly distributed on the surface of the failure zone: $q=\frac{P_{C}}{\pi ab}=\frac{\xi }{\pi a^{2}}P_{C}$, where $\xi =\frac{a}{b}$.

The relationship between the internal force, bending moment, and displacement can be obtained by substituting the strain component and stiffness matrix of [*A_i_*], [*B_i_*], and [*D_i_*] into Equation (3). Then, the internal force and bending moment are substituted into Equation (12) to obtain the relationship between the displacement and *P_C_* (Equations (13)–(17)). C*_j_* is the material performance coefficient of the undrilled material.

(13) $$u_{1}=P_{C}C_{1}a$$

(14) $$u_{2}=P_{C}C_{4}a$$

(15) $$\omega_{0}=P_{C}C_{3}a^{2}$$

(16) $$v_{1}=P_{C}C_{2}a$$

(17) $$v_{2}=P_{C}C_{5}a$$

The deflection of the undrilled layer material is obtained by deriving the diameter of the elliptical long axis in Equation (15):

(18) $$d\omega_{0}=2aP_{C}C_{3}da$$

### 4.2. Solution of Elastic Strain Energy dU of Undrilled Material

According to classical plate theory, the strain energy of the plate model can be obtained via the following equation:

(19) $$U=\frac{1}{2}\int \sigma :\;\varepsilon dV=\frac{1}{2}\int \sigma :\;\left(\left\{\varepsilon^{m}\right\}+\left\{\varepsilon^{t}\right\}\right)dV$$

Here, the relationship between stress and strain under thermal–mechanical coupling is:

(20) $$\left[\begin{array}{l} \sigma_{x}\\ \sigma_{y}\\ \tau_{xy} \end{array}\right]=\left[\begin{array}{ccc} {\bar{Q}}_{11} & {\bar{Q}}_{12} & {\bar{Q}}_{16}\\ {\bar{Q}}_{12} & {\bar{Q}}_{22} & {\bar{Q}}_{26}\\ {\bar{Q}}_{16} & {\bar{Q}}_{26} & {\bar{Q}}_{66} \end{array}\right]\left[\begin{array}{l} \varepsilon_{x}\\ \varepsilon_{y}\\ \varepsilon_{z} \end{array}\right]=\left[\begin{array}{ccc} {\bar{Q}}_{11} & {\bar{Q}}_{12} & {\bar{Q}}_{16}\\ {\bar{Q}}_{12} & {\bar{Q}}_{22} & {\bar{Q}}_{26}\\ {\bar{Q}}_{16} & {\bar{Q}}_{26} & {\bar{Q}}_{66} \end{array}\right]\left(\left[\begin{array}{l} \varepsilon_{x}^{m}\\ \varepsilon_{y}^{m}\\ \gamma_{xy}^{m} \end{array}\right]+\left[\begin{array}{l} \alpha_{x}\\ \alpha_{y}\\ \alpha_{xy} \end{array}\right]\Delta T\right)$$

where $\varepsilon_{{}_{x}}^{}$ is the total strain $\varepsilon_{{}_{x}}^{m}$ is the strain caused by mechanical force; and $\alpha_{{}_{x}}^{}$ is the coefficient of thermal expansion.

Equation (21) is obtained by expanding Equation (20) and substituting the expanded Equation (20) into Equation (19):

(21) $$\begin{array}{l} U=\frac{1}{2}\iint_{S}(A_{11}\varepsilon_{x}^{2}+2A_{12}\varepsilon_{x}\varepsilon_{y}+2A_{16}\varepsilon_{x}\varepsilon_{xy}+2B_{11}\varepsilon_{x}\tau_{x}\\ +2B_{12}\varepsilon_{x}\tau_{y}+\ldots +D_{26}\tau_{xy}^{2})dxdy-\frac{1}{2}\iint_{S}(N_{x}^{T}\frac{\partial u}{\partial x}+N_{y}^{T}\frac{\partial v}{\partial y}+\\ N_{xy}^{T}(\frac{\partial u}{\partial y}+\frac{\partial v}{\partial x}))dxdy+\frac{1}{2}\iint_{S}(M_{x}^{T}\frac{\partial^{2}\omega }{\partial x^{2}}+M_{y}^{T}\frac{\partial^{2}\omega }{\partial y^{2}}+\\ 2M_{xy}^{T}\frac{\partial^{2}\omega }{\partial x\partial y})dxdy+\frac{1}{2}\iint_{S}\int_{-\frac{h}{2}}^{\frac{h}{2}}(Q_{{}_{11}}^{k}{\left(\varepsilon_{x}^{tk}\right)}^{2}+Q_{{}_{22}}^{k}{\left(\varepsilon_{y}^{tk}\right)}^{2}+4Q_{{}_{66}}^{k}{\left(\varepsilon_{xy}^{tk}\right)}^{2}\\ +4Q_{{}_{12}}^{k}\varepsilon_{xy}^{tk}\varepsilon_{y}^{tk}+4Q_{{}_{16}}^{k}\varepsilon_{x}^{tk}\varepsilon_{xy}^{tk}+4Q_{{}_{26}}^{k}\varepsilon_{y}^{tk}\varepsilon_{xy}^{tk})dzdxdy \end{array}s:\frac{x^{2}}{a^{2}}+\frac{y^{2}}{b^{2}}-1\leq 0$$

Assuming that the temperature difference between the drilling temperature and the drilling ambient temperature is 0, $\left\{\varepsilon_{}^{t}\right\}=0$, and the strain energy generated by pure mechanical strain is:

(22) $$\begin{array}{l} U_{m}=\frac{1}{2}\iint_{S}(A_{11}\varepsilon_{x}^{2}+2A_{12}\varepsilon_{x}\varepsilon_{y}+2A_{16}\varepsilon_{x}\varepsilon_{xy}+\\ 2B_{11}\varepsilon_{x}\tau_{x}+2B_{12}\varepsilon_{x}\tau_{y}+\ldots +D_{26}\tau_{xy}^{2})dxdy \end{array}$$

Pure mechanical strain can be obtained by substituting Equations (13)–(17) into Equations (4) and (9):

(23) $$\varepsilon_{x}=P_{C}[C_{1}(1-\frac{x^{2}}{a^{2}}-\frac{y^{2}}{b^{2}})-\frac{2}{a^{2}}(C_{1}x^{2}+C_{4}\xi xy)]$$

(24) $$\varepsilon_{y}=P_{C}[C_{2}\xi (1-\frac{x^{2}}{a^{2}}-\frac{y^{2}}{b^{2}})-\frac{2}{b^{2}}(C_{5}xy+C_{2}\xi y^{2})]$$

(25) $$\begin{array}{l} \varepsilon_{xy}=P_{C}[(C_{4}\xi +C_{5})(1-\frac{x^{2}}{a^{2}}-\frac{y^{2}}{b^{2}})-\\ \frac{2}{a^{2}}(C_{5}x^{2}+C_{2}\xi xy)-\frac{2}{b^{2}}(C_{1}xy+C_{4}\xi y^{2})] \end{array}$$

(26) $$\tau_{x}=4P_{C}C_{3}(1-\frac{3x^{2}}{a^{2}}-\frac{y^{2}}{b^{2}})$$

(27) $$\tau_{y}=4P_{C}C_{3}\xi^{2}(1-\frac{x^{2}}{a^{2}}-\frac{3y^{2}}{b^{2}})$$

(28) $$\tau_{xy}=-\frac{16P_{C}C_{3}}{b^{2}}xy$$

By substituting Equations (23)–(28) into Equation (22), the strain energy of undrilled laminates caused by mechanical force can be obtained when the drilling temperature difference is 0:

(29) $$U_{m}=KP_{C}^{2}a^{2}$$

where:

(30) $$\begin{array}{l} K=\frac{\pi }{12}\left\{\begin{array}{l} \frac{A_{11}}{\xi }(3C_{1}^{2}+C_{4}^{2})+2A_{12}(C_{1}C_{2}+C_{4}C_{5})+\\ A_{22}(3C_{2}^{2}+C_{5}^{2})+\frac{2A_{11}}{\xi }(2\xi C_{1}C_{4}+3C_{1}C_{5}+C_{2}C_{4}) \end{array}\right\}\\ +2A_{26}(3\xi C_{2}C_{4}+2C_{2}C_{5}+\xi C_{1}C_{5})+\frac{A_{66}}{\xi }\left[\begin{array}{l} \xi {\left(C_{1}+C_{2}\right)}^{2}+3\xi^{2}C_{4}^{2}\\ +3C_{5}^{2}+2\xi C_{4}C_{5} \end{array}\right]\\ +\frac{24B_{11}}{\xi }C_{1}C_{3}+8B_{12}C_{3}(\xi C_{1}+C_{2})+24B_{16}C_{3}(C_{4}+\frac{C_{5}}{\xi })\\ +24B_{22}\xi^{2}C_{2}C_{3}+24B_{26}\xi C_{3}(C_{5}+\xi C_{4})+16B_{66}C_{3}(\xi C_{1}+C_{2})\\ +\frac{16D_{11}C_{3}^{2}}{\xi }(3D_{11}+2\xi^{2}D_{12}+3\xi^{4}D_{22}+4\xi^{2}D_{66}) \end{array}$$

Referring to the solution of thermal strain in Saoudi [9], when the temperature difference between the drilling temperature and the drilling ambient temperature is not zero, the total strain energy including thermal strain caused by the undrilled laminate is as follows:

(31) $$\begin{array}{l} U=U_{m}+U_{t}=KP_{C}^{2}a^{2}+\frac{1}{2}\iint_{S}(N_{x}^{T}\frac{\partial u}{\partial x}+N_{y}^{T}\frac{\partial v}{\partial y}+N_{xy}^{T}(\frac{\partial u}{\partial y}+\frac{\partial v}{\partial x}))dxdy\\ +\frac{1}{2}\iint_{S}(M_{x}^{T}\frac{\partial^{2}\omega }{\partial x^{2}}+M_{y}^{T}\frac{\partial^{2}\omega }{\partial y^{2}}+2M_{xy}^{T}\frac{\partial^{2}\omega }{\partial x\partial y})dxdy+\\ \frac{1}{2}\iint_{S}\int_{-\frac{h}{2}}^{\frac{h}{2}}(Q_{{}_{11}}^{k}{\left(\varepsilon_{x}^{tk}\right)}^{2}+Q_{{}_{22}}^{k}{\left(\varepsilon_{y}^{tk}\right)}^{2}+4Q_{{}_{66}}^{k}{\left(\varepsilon_{xy}^{tk}\right)}^{2}+\\ 4Q_{{}_{12}}^{k}\varepsilon_{xy}^{tk}\varepsilon_{y}^{tk}+4Q_{{}_{16}}^{k}\varepsilon_{x}^{tk}\varepsilon_{xy}^{tk}+4Q_{{}_{26}}^{k}\varepsilon_{y}^{tk}\varepsilon_{xy}^{tk})dzdxdy \end{array}$$

The basic assumptions of classical laminated plate theory are:

(32) $$\varepsilon_{yz}=\varepsilon_{xz}=\varepsilon_{{}_{yz}}^{t}=\varepsilon_{{}_{xz}}^{t}=0$$

(33) $$\sigma_{z}=0$$

Then, in Equation (31):

(34) $$\iint_{S}(N_{x}^{T}\frac{\partial u}{\partial x}+N_{y}^{T}\frac{\partial v}{\partial y}+N_{xy}^{T}(\frac{\partial u}{\partial y}+\frac{\partial v}{\partial x}))dxdy=0$$

(35) $$\iint_{S}(M_{x}^{T}\frac{\partial^{2}\omega }{\partial x^{2}}+M_{y}^{T}\frac{\partial^{2}\omega }{\partial y^{2}}+2M_{xy}^{T}\frac{\partial^{2}\omega }{\partial x\partial y})dxdy=0$$

(36) $$\begin{array}{l} \iint_{S}\int_{-\frac{h}{2}}^{\frac{h}{2}}(Q_{{}_{11}}^{k}{\left(\varepsilon_{x}^{tk}\right)}^{2}+Q_{{}_{22}}^{k}{\left(\varepsilon_{y}^{tk}\right)}^{2}+4Q_{{}_{66}}^{k}{\left(\varepsilon_{xy}^{tk}\right)}^{2}+\\ 4Q_{{}_{12}}^{k}\varepsilon_{xy}^{tk}\varepsilon_{y}^{tk}+4Q_{{}_{16}}^{k}\varepsilon_{x}^{tk}\varepsilon_{xy}^{tk}+4Q_{{}_{26}}^{k}\varepsilon_{y}^{tk}\varepsilon_{xy}^{tk})dzdxdy\\ =\frac{\pi a^{2}}{\xi }D^{*} \end{array}$$

where:

(37) $$\begin{array}{l} D^{*}=({\bar{D}}_{11}+{\bar{D}}_{22}+4{\bar{D}}_{12}+4{\bar{D}}_{16}\\ +4{\bar{D}}_{26}+4{\bar{D}}_{66}){\left(\Delta T\right)}^{2} \end{array}$$

where Δ*T* is the temperature difference between the drilling temperature and the ambient temperature.

(38) $${\bar{D}}_{ij}\sum_{k=1}^{n}(\frac{z_{k}^{3}-z_{k-1}^{3}}{3}){\bar{Q}}_{ij}^{k}\alpha_{i}^{k}\alpha_{j}^{k};i,j=1,2,6$$

Then, the calculation equation of undrilled materials’ strain energy under mechanical-thermal coupling can be deduced:

(39) $$U=KP_{C}^{2}a^{2}+\frac{\pi a^{2}}{\xi }D^{*}$$

From the derivation of Equation (39), we can obtain:

(40) $$dU=2(KP_{C}^{2}a+\frac{\pi a}{\xi }D^{*})da$$

### 4.3. Solution of Fracture Randomness

Through the analysis of the results of a three-dimensional drilling finite element analysis, it can be seen that, when the drill bit is drilled at the exit, the delamination failure mode is type I/III mixed mode, so the coupling of I and III cracks should be considered in the analytical model of *P_C_*. According to the BK damage criterion:

(41) $$\frac{G_{I}}{G_{IC}}+\frac{G_{III}}{G_{IIIC}}=1$$

Then,

(42) $$G_{C}=rG_{IIIC}+(1-r)G_{IC}$$

where *r* is the mixing coefficient of the fracture toughness of type I/III cracks and $r={\left(\frac{G_{III}}{G_{I}+G_{III}}\right)}^{\eta }$. This can be solved via the conjugate gradient method according to inverse problem theory.

When substituting the Gc, dA, dw, and *dU* obtained above into the critical layered energy balance relationship, the analytical equation of the *P_C_* can be obtained as follows:

(43) $$P_{C}=\sqrt{\frac{\pi ((rG_{IIIC}+(1-r)G_{IC})+D^{*})}{\xi (C_{3}-K)}}$$

**Figure 9:**
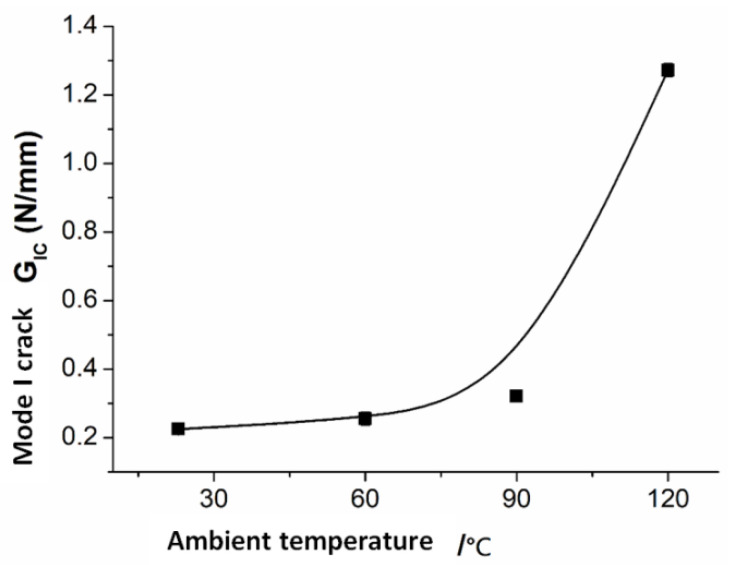
Mode I crack fracture toughness vs. ambient temperature.

**Figure 10:**
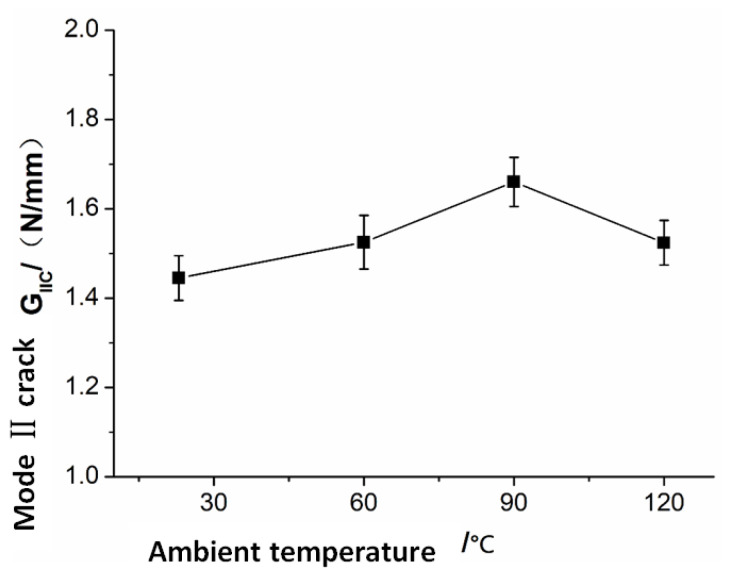
Mode II crack fracture toughness vs. ambient temperature.

The temperature-dependent parameters in the equation are G*_IC_*, G*_IIIC_*, K, *C_j_*, and D*. The mechanical parameters of *G_IC_* that vary with temperature are obtained via the *ASTM D5528-01* standard [17], the experimental results of *G_IC_* are shown in Figure 9, and a detailed experimental process is shown in [18]; the mechanical parameters of *G_IIIC_* that vary with temperature are obtained from the measurement results of *G_IIC_* [19], the mechanical parameters of *G_IIC_* that vary with temperature are obtained via the *ASTM D7905M-14* standard [20], the experimental results of *G_I__I__C_* are shown in Figure 10, and the detailed experimental process is shown in Appendix A; the values of *K* are calculated via Equation (30); the calculation methods of *C_j_* are from [8].

**Figure 11:**
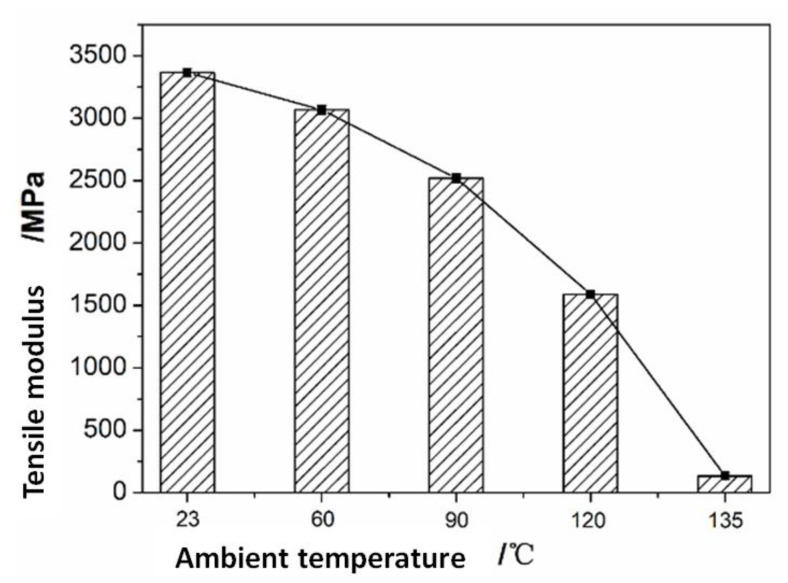
Resin modulus vs. ambient temperature.

**Figure 12:**
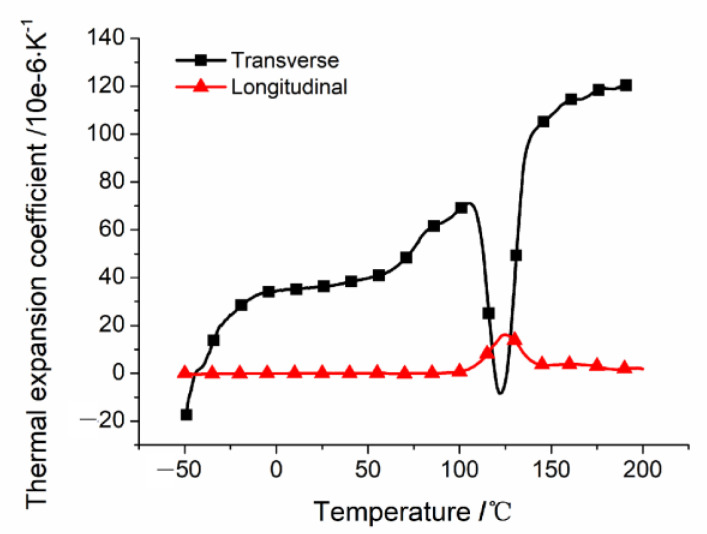
Change curve of thermal expansion coefficient of one-way plate with temperature.

Both *K* and *C_j_* are calculated by the combination of the [*A_i_*], [*B_i_*], and [*D_i_*] stiffness matrices. As the mechanical properties of carbon fibers hardly change with a change in drilling temperature in this temperature range, the parameters affecting the values of the [*A_i_*], [*B_i_*], and [*D_i_*] stiffness matrices are calculated using the modulus of the resin matrix with temperature, which is obtained via the tensile test of the resin at different ambient temperatures (Figure 11). The detailed experimental process is shown in [18]; in addition to the stiffness matrix of [*A_i_*], [*B_i_*], and [*D_i_*], the value of D* at different temperatures is also affected by the thermal expansion coefficient. The thermal expansion coefficients of the unidirectional CFRP were measured using a dilatometer (NETZSCH DIL 402C). The test sample laying method was [0]_40_, the sample size was 10 mm × 10 mm × 5 mm, and the measuring temperature range was −50–200 °C. The thermal expansion coefficients of the unidirectional CFRP in the longitudinal and transverse directions are shown in Figure 12.

### 4.4. The Solution of the Layered Area

Assuming that the shape of the exit layer is an ellipse, the area of the layer is the area of the ellipse minus the area of the ideal hole. The assumptions are:

(44) $$\frac{a}{b}=\frac{\left(a+da\right)}{\left(b+db\right)}=\frac{da}{db}=cons\tan t=\xi$$

where ξ is an ellipse ratio. Then, the differential of the stratified area is:

(45) $$dA=\pi (a+da)(b+db)-\pi ab=2\pi bda=\frac{2\pi a}{\xi }da$$

## 5. Critical Axial Force Verification Test

### 5.1. Experimental Setup

To verify the accuracy of the drilling *P_C_* model derived above, a temperature-controlled *P_C_* experiment must be carried out. However, due to the influence of the process parameters on the drilling temperature and drilling force, it is difficult to obtain a *P_C_* that only produces delamination at different drilling temperatures through drilling tests. In addition, the experimental data from past studies on drilling *P_C_* cannot be referenced because the influence of drilling temperature is not considered, but the experimental method can be referenced. In previous studies, a static compression experiment was used to measure the *P_C_*.

To verify the theoretical model introduced in the previous section, this section draws on this test method to design a *P_C_* equivalent test at different ambient temperatures. However, because the *P_C_* experiment can only approximately simulate the axial force generated by the downward feed of the drill bit and cannot simulate the torque caused by the rotation of the drill bit, the failure mode of the exit stratification of the *P_C_* experiment is the only mode I open failure.

The CFRP composite used in this study was a carbon T300/epoxy unidirectional prepreg with a ply thickness of 0.125 mm. The paving sequence of materials used in the experiment is [0/45/90/−45]_4S_. The reason why we use [0/45/90/−45]_4S_ to verify the analytical mode is that a thick layer can form better-quality blind holes. After laying the plate preform manually, we put it into the autoclave for heating and curing. The curing conditions are heating to 80 °C, holding for 30 min, then pressurizing to 0.5 MPa, heating to 120 °C, holding for 90 min, and finally cooling in a furnace. During the experiment, non-drilled conical blind holes with a thickness of 1–6 layers are prefabricated on the tested sample. Due to the taper of the drill, when the number of layers is small, the horizontal edge of the drill drills the blind holes. To ensure that drilling stratification does not occur during the prefabrication of blind holes, a back plate is placed under the laminated plate to be drilled during drilling to increase the exit stiffness. The spindle speed is 4000 rpm, and the feed rate is 0.03 mm/r.

**Figure 13:**
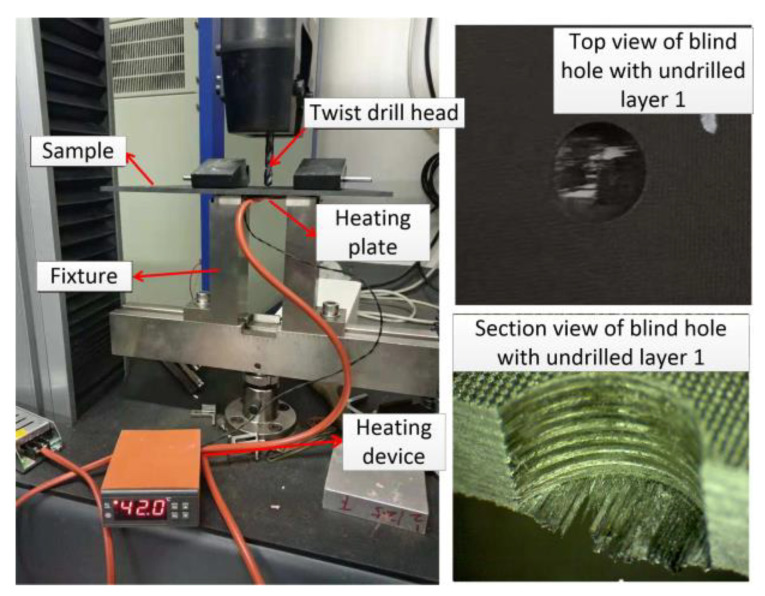
Experimental platform for critical axial force testing.

Figure 13 shows the *P_C_* testing bench at different ambient temperatures. During the experiment, the sample was fixed on the platform of the universal testing machine. The exit temperature of the blind hole was locally heated by a silica gel heater. The temperature of the heater was set to 23 °C, 60 °C, 90 °C, and 120 °C, respectively. The experimental pressure head was a carbide drill bit with a diameter of 8 mm. The compression speed was set to 2 mm/min during the test.

### 5.2. Result Discussion

**Figure 14:**
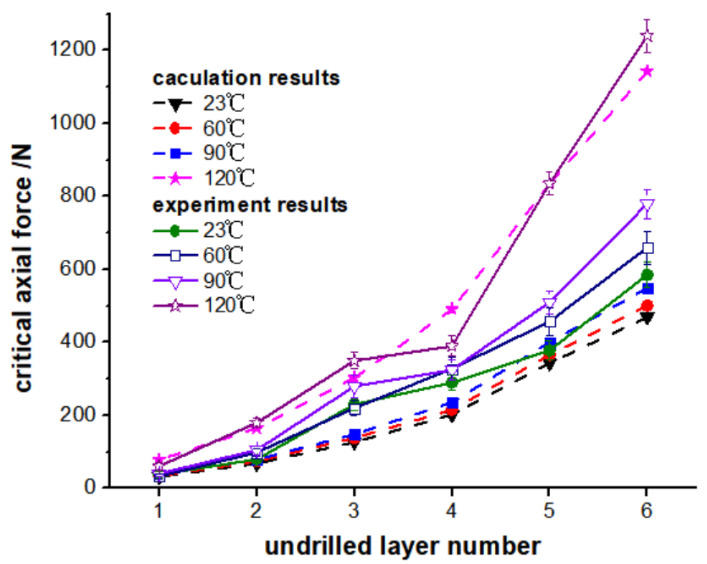
Comparison of the theoretical and experimental solutions of critical axial force.

Figure 14 shows a comparison between the *P_C_* results predicted by Equation (45) and the experimental results under different drilling ambient temperatures. It can be seen from Figure 14 that the theoretically predicted *P_C_* value is in good agreement with the experimental value when the drilling temperature is 120 °C, and there are some errors when the drilling temperature is 23 °C, 60 °C, or 90 °C. There are two reasons for this error. Firstly, the influence of the geometry of the drill bit on the drilling axial force distribution is not considered in this *P_C_* model. Secondly, when measuring the fracture toughness, the influence of different ply angles is not considered.

However, both the prediction results and the experimental results show that the drilling temperature has a great impact on the critical axial force, and the critical axial force increases with an increase in the drilling temperature. Additionally, the change trend of the curve is consistent when the drilling temperature is 23 °C, 60 °C, and 90 °C. From the general trend, it can be seen that the *P_C_* of CFRP drilling delamination is greatly influenced by the mechanical properties of the CFRP. When the drilling temperature is not greater than the glass transition temperature range of the material itself (about 120 °C), due to the increase in fracture toughness, the *P_C_* increases with an increase in drilling temperature.

## 6. Conclusions

This paper focuses on the influence of the temperature-dependent characteristics of CFRPs’ mechanical properties on the *P_C_* of drilling delamination damage. First, the failure mode of drilling delamination is qualitatively analyzed through a three-dimensional finite element model. Second, a new drilling *P_C_* model, which considers the temperature-dependence of CFRPs’ mechanical properties and the failure modes of CFRP drilling delamination, is established based on the classical drilling critical force mechanics model. The influence of the change in CFRPs’ mechanical properties on the *P_C_* under different drilling temperatures is analyzed, and the model is verified at different temperatures. The main findings are as follows:

1. Through the finite element simulation results, it can be found that the failure mode of drilling exit delamination is mainly the I/III mixed crack failure mode;
2. Due to the influence of the temperature-dependent characteristics of CFRP mechanical properties, when the drilling temperature changes, the *P_C_* is not a fixed value. The drilling temperature has a great impact on *P_C_* when the temperature is lower than the glass transition temperature. The fracture toughness increases with an increase in drilling temperature, and the value of *P_C_* increases with an increase in drilling temperature.

## Figures and Tables

**Table 1:** Summary of *P_C_* Prediction Models.

Reference	Formula of *P_C_*
Ojo [4]	$P_{C}=\sqrt{\frac{\pi G_{C}}{\xi \left(C_{3}/3-K_{m}-K_{s}\right)}}$
Hocheng [5]	$P_{C}=\pi \sqrt{\frac{8G_{IC}Eh^{3}}{3-\left(1-\upsilon^{2}\right)}}$
Lachuad [6]	$P_{C}=8\pi \sqrt{\frac{G_{IC}D}{1/3-\left(D^{\prime }/8D\right)}}$
Jain [7]	$P_{C}=3\pi {\left(\frac{D_{22}}{D_{11}}\right)}^{1/4}\sqrt{G_{IC}D_{{}_{C}}^{*}}$
Zhang [8]	$P_{C}=\sqrt{\frac{\pi G_{IC}}{\xi \left(C_{3}-K\right)}}$
Saoudi [9]	$P_{C}=\sqrt{\frac{\pi \left(K^{*}+G_{IC}\right)}{\xi \left(C_{3}/3-K\right)}}$

**Table 2:** Mechanical properties of CFRP at room temperature.

Elastic Property	Numerical Value	Damage Characteristic	Numerical Value
Longitudinal tensile modulus, *E_11_* (MPa)	137,000	Longitudinal tensile strength, *X_T_* (MPa)	2000
Transverse tensile modulus, *E_22_* (MPa)	9000	Longitudinal compressive strength, *X_C_* (MPa)	1150
Axial tensile modulus, *E_33_* (MPa)	9000	Transverse tensile strength, *Y_T_* (MPa)	60
Poisson’s ratio, $\upsilon_{12}$	0.28	Transverse compressive strength, *Y_C_* (MPa)	152
Poisson’s ratio, $\upsilon_{13}$	0.28	Longitudinal shear strength, *S_L_* (MPa)	75
Poisson’s ratio, $\upsilon_{23}$	0.4	Transverse shear strength, *S_T_* (MPa)	76
Shear modulus, *G_12_* (MPa)	3780	Glass transition temperature *Tg* (°C)	117
Shear modulus, *G_13_* (MPa)	6000		
Shear modulus, *G_23_* (MPa)	6000		
Density, *ρ* (t/mm^3^)	1.79 × 10^−9^		

**Table 3:** Mechanical properties of CFRP interlamination at room temperature.

Elastic Property	Numerical Value	Damage Characteristic	Numerical Value
Elastic modulus in the normal direction, $K_{\mathrm{nn}}$ (MPa)	14,000	Normal strength, $t_{n}^{0}$ (MPa)	70
Elastic modulus in shear I direction, $K_{\mathrm{ss}}$ (MPa)	26,000	Shear I direction strength, $t_{s}^{0}$ (MPa)	60
Elastic modulus in shear direction II, $K_{\mathrm{tt}}$ (MPa)	26,000	Shear II direction strength, $t_{t}^{0}$ (MPa)	60
Density, *ρ* ($t/mm^{3}$)	1.79 × 10^−9^	Mode I crack fracture toughness, $G_{Ic}$ ($J/m^{2}$)	220
		Mode II crack, $G_{IIc}$ ($J/m^{2}$)	1445
		Mode III crack, $G_{IIIc}$ ($J/m^{2}$)	1445

**Figure A1:**
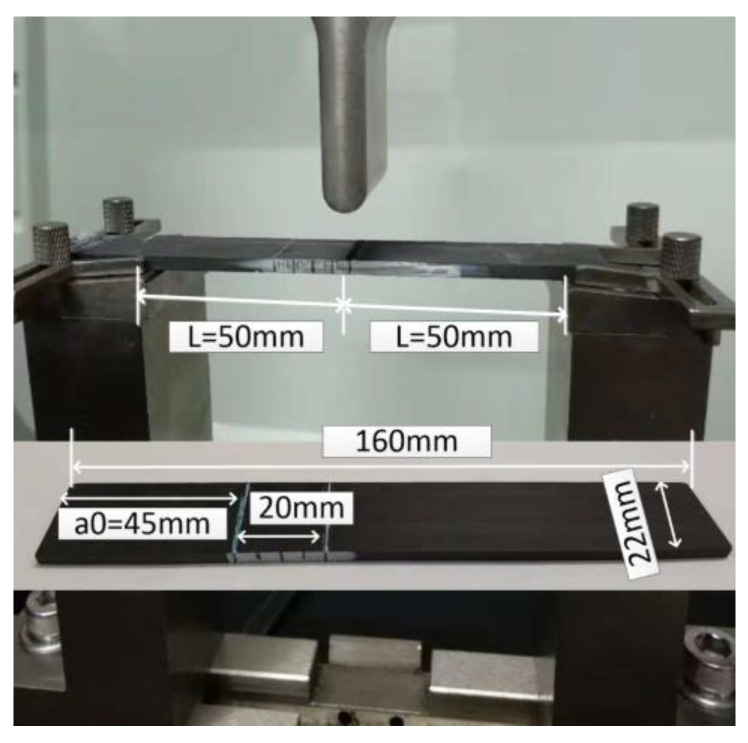
Detailed experiments and sample settings.

**Figure A2:**
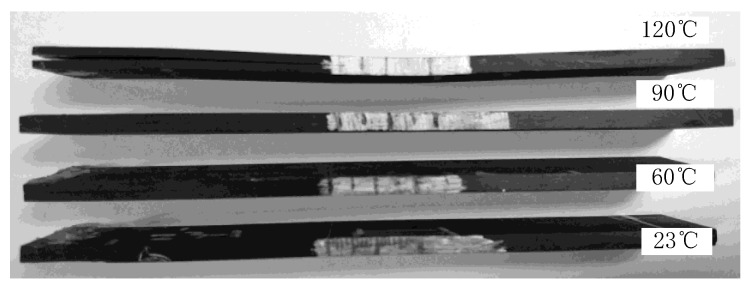
Representative samples tested at different ambient temperatures.

**Figure A3:**
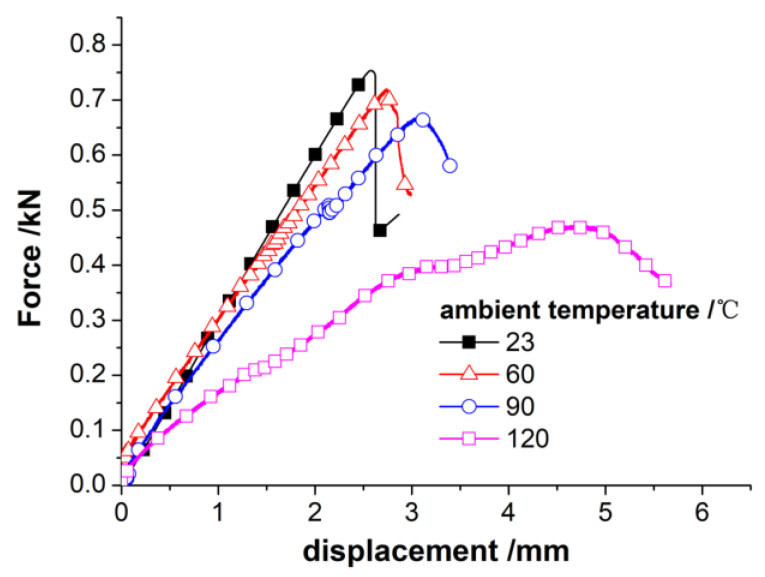
Representative curves tested at different ambient temperatures.

## Data Availability

The data presented in this study are available on request from the corresponding authors.

## Appendix A

### Mode II Interlaminar Fracture Toughness Testing

The influence of temperature on *G_IIC_* was tested using the three-point bending test, according to the standard of *ASTM D7905M-14* [20]. The sample dimensions were 160 mm × 22 mm × 3.5 mm. The paving method is [0]_28_, and the initial delamination length was 45 mm. All tests were performed at a speed of 2 mm/min. Set the test temperature as 23 °C, 60 °C, 90 °C, and 120 °C, respectively. Five tests were performed at each temperature. Detailed experiments and sample settings are shown in Appendix A Figure A1.

The test samples measured at different ambient temperatures are shown in Appendix A Figure A2. The force/displacement curves measured at different ambient temperatures are shown in Appendix A Figure A3. The following equation was used to calculate *G_IIC_*:

(A1) $$G_{IIC}=\frac{9P\delta a^{2}}{2b\left(2L^{3}+3a^{3}\right)}$$

where *p* is the load (N); *δ* is the load point displacement (mm); *b* is the specimen width (mm); *a* is the delamination length (mm); and *L* is half the span (mm). Combined with the force/displacement curve and Formula (2), the Mode II interlaminar fracture toughness at different ambient temperatures can be calculated. The results are shown in Figure 10.
